# Early Life Added Sugars and Associated Appetite, Satiety, Growth and Adiposity in the First 2 Years of Life

**DOI:** 10.3390/nu18050833

**Published:** 2026-03-04

**Authors:** Sofía Barragán-Vázquez, Ivonne Ramírez-Silva, Gabriela Olvera-Mayorga, Mónica Ancira-Moreno, Juan A. Rivera Dommarco, Alejandra Cantoral, Laura Ávila-Jimenez, María Alejandra Terrazas Meraz, Santiago Andrés Henao Moran, Diane Threapleton

**Affiliations:** 1 Center for Nutrition and Health Research, National Institute of Public Health, Av. Universidad 655, Col. Santa Maria Ahuacatitlan, Cerrada Los Pinos y Caminera, Cuernavaca 62100, Mexico; s.barragan@leeds.ac.uk (S.B.-V.); or gabrielaolveramg@gmail.com (G.O.-M.); 2 Nutritional Epidemiology Group, School of Food Science & Nutrition, University of Leeds, Woodhouse Ln, Woodhouse, Leeds LS2 9 JT, UK; d.e.threapleton@leeds.ac.uk; 3 Nutrition Academic Area, Institute of Health Sciences, Autonomous University of the State of Hidalgo, San Agustin Tlaxiaca 42160, Mexico; 4 Health Department, Iberoamerican University, Mexico City 01219, Mexico; monica.ancira@ibero.mx (M.A.-M.); acantoral@hotmail.com (A.C.); 5 Center for Population Health Research, National Institute of Public Health, Av. Universidad 655, Col. Santa Maria Ahuacatitlan, Cerrada Los Pinos y Caminera, Cuernavaca 62100, Mexico; jrivera@insp.mx; 6 Coordinación Auxiliar Médica de Investigación en Salud, Delegación Estatal Morelos, Instituto Mexicano del Seguro Social, Cuernavaca 62000, Mexico; laura.avilaj@gmail.com; 7 Faculty of Nutrition, Autonomous University of the State of Morelos, Cuernavaca 62350, Mexico; maria.alejandra@uaem.mx; 8 “Salvador Zubiran” National Institute of Medical Sciences and Nutrition, Mexico City 14080, Mexico; henaosa@yahoo.com

**Keywords:** added sugars, adiposity, child, preschool, infant, breastfeeding

## Abstract

**Introduction**: Added sugar (AS) intake has been linked to chronic diseases, yet evidence in children under 2 years remains limited. **Aim**: Characterise AS intake in children ≤ 2 years with associated appetite, satiety, growth, adiposity, and breastfeeding duration. **Methods**: We analysed data from 248 mother-child pairs from the MAS-Lactancia birth cohort. Intake of AS and energy was estimated using data from 24 h dietary recalls. AS intake was classified in tertiles as low (0 g), medium (0.01–6.96 g), and high (>6.96 g). Major food group contributors to AS intake were identified. Appetite and satiety indicators were measured using the Child Eating Behaviour Questionnaire. Adiposity was evaluated using body mass index-for-age Z score, waist circumference, and skinfold thickness. Growth was assessed using length-for-age Z score (ZLA). Linear mixed-effects models were fitted. **Results**: AS intake and its contribution to total energy increased with age. Major contributors to AS intake were infant formulas, table sugars, and sweet baked goods. Longer exclusive and continued breastfeeding were associated with lower AS intake. Compared to low intake, children with high AS intake had higher scores for emotional overeating (β = 0.58, 95% CI: 0.04, 1.12) and food fussiness (β = 1.45, 95% CI: 0.38, 2.53). High AS intake was also associated with lower ZLA (β = −0.17 z, 95% CI: −0.32, −0.01) and higher waist circumference (β = 2.02 cm, 95% CI: 1.32, 2.73). **Conclusions**: Among children ≤ 2 years, AS intake ≥ 7 g/d was associated with suboptimal growth, central adiposity, and less favourable eating behaviours. Longer breastfeeding duration may protect against AS exposure.

## 1. Introduction

Added sugars (AS) are sugars incorporated into foods and beverages during processing or preparation, excluding naturally occurring sugars (e.g., lactose in milk and fructose in fruit). These sugars are often used as food ingredients to enhance palatability, improve texture, and prolong shelf life [1]. A growing body of evidence shows that intake of AS is associated with the development of obesity, cardiovascular disease, hypertension, obesity-related cancers, and dental caries [2]. Therefore, international and country-level recommendations advise that children under 2 years of age should ideally avoid AS [2,3]. Some guidelines also refer to free sugars [4], which include added sugars and those that are naturally present in juiced or pureed fruit and vegetables [1], with suggested intake limits of ≤5% of total energy.

The first 1000 days of life represent a critical period for the development of sensory perception and food preferences. Flavour perception begins in utero and continues throughout early-life milk feeding (breastfeeding or infant formula) and beyond [5]. After birth, infants display characteristic taste preferences: sweet and umami flavours elicit positive responses, whereas bitter and sour flavours tend to provoke negative reactions [6]. However, these preferences are modifiable and are shaped by a range of social and environmental factors, allowing infants to accept and eventually prefer foods available within their surroundings [5,6].

Stimulation of sweet taste receptors activates reward centers in the brain associated with pleasure through mechanisms similar to those observed with alcohol and drugs [5]. Also, the effects of different sugars on food intake appear to vary at the hypothalamic level. Evidence shows that the administration of glucose suppresses food intake, whereas fructose increases it [7]. In addition to influencing the hypothalamic appetite and satiety pathways, sugar also affects reward systems due to its addictive, palatable, and rewarding properties, which may alter hypothalamic responses and promote compulsive eating [7]. Higher sugar intake has been reported to reduce overall nutrient density by displacing more nutrient-rich foods while providing substantial energy, a pattern that may contribute to increased adiposity and suboptimal linear growth during early childhood [8,9]. These early neurobiological responses to sweet taste may therefore influence food preferences, sugar consumption patterns, and long-term dietary behaviors, underscoring the importance of limiting AS during infancy and early childhood.

In Mexico, according to data from the National Health and Nutrition Survey (ENSANUT), in 2012 the consumption of AS among children aged 1 to 4 years contributed 12.1% of total dietary energy intake, and 52.5% of AS consumption came from sugar-sweetened beverages [10]. Also, around 35% of children aged 6–23 months and 12% of children < 6 months of age consumed non-dairy sugar-sweetened beverages [11]. Mexico represents a particularly relevant context for examining early AS intake given the high availability and widespread consumption of sugar-sweetened beverages, the early introduction of sweetened drinks during infancy, and the country’s persistently high rates of childhood overweight and obesity [12].

Despite extensive research on the impacts of added sugar in older children and adult populations, we lack data in this critical early life period. Evidence regarding its effects in children under 2 years of age remains limited, and appetite- and satiety-related behaviors in relation to added sugar intake have been particularly underexplored. Therefore, this study aims to characterize AS intake during the first two years of life and to examine its association with indicators of appetite, satiety, growth, adiposity, and breastfeeding duration.

## 2. Materials and Methods

### 2.1. Design and Study Population

**Figure 1:**
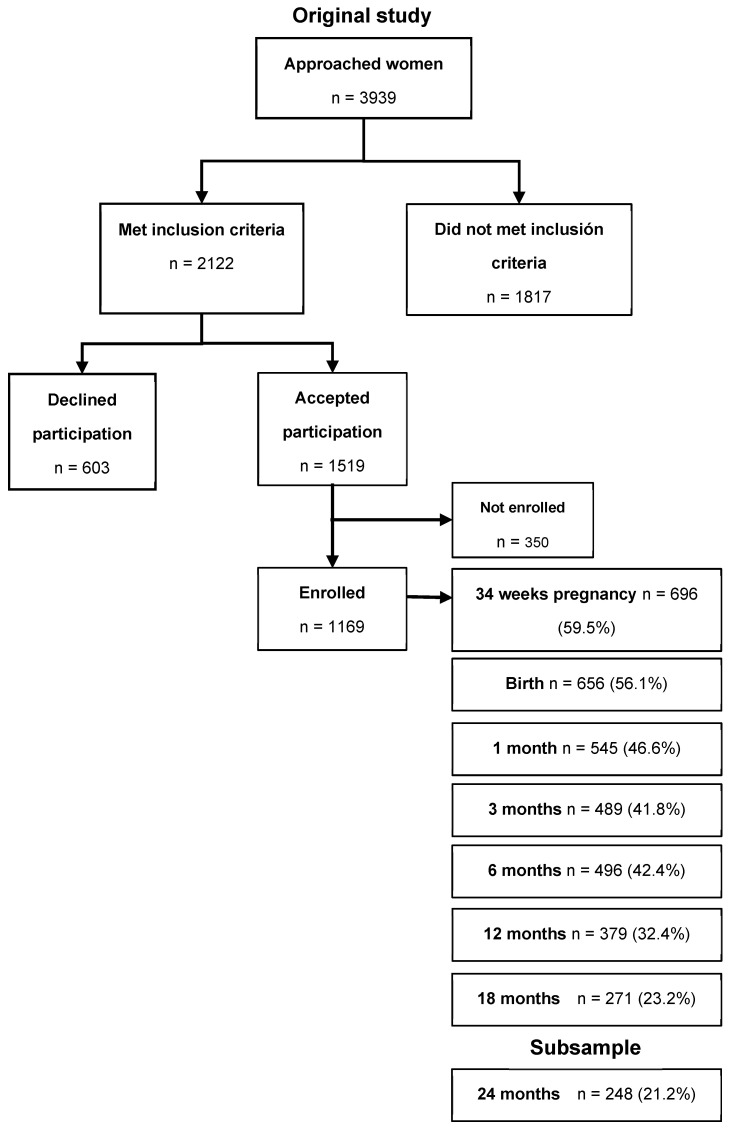
Study Sample Selection from the MAS-Lactancia Cohort.

Data from 248 mother–child pairs enrolled in the MAS-Lactancia cohort [13] were analysed. The subsample had sociodemographic, dietary, anthropometric, appetite, and satiety information collected from birth to 24 months of age (Figure 1), representing 21.2% of the total cohort.

The primary aim of the original study was to examine the association between infant feeding practices and fat-mass-and-obesity-associated gene (FTO), adiponectin, and leptin polymorphisms with growth and adiposity during the first 60 months of life, and to explore the mediating role of appetite and satiety indicators in these associations. The study began in 2016 with the recruitment of pregnant women from two clinics of the Mexican Social Security Institute (IMSS in Spanish) in Cuernavaca, Morelos. Eligible participants were women aged 18–39 years who were between 16 and 22 weeks of gestation. Women were excluded if they had a diagnosis of hypertension, preeclampsia, renal, hepatic, or cardiovascular diseases; multiple pregnancies; preterm birth (<37 weeks); substance abuse; or if their infants had congenital conditions that could affect growth and feeding (e.g., cleft lip and palate, food allergies) or physical malformations that could interfere with anthropometric measurements. All recruited women were offered breastfeeding counselling beginning at 34 weeks of gestation through in-person sessions and printed educational materials as part of an intervention to increase exclusive breastfeeding prevalence in the cohort. The study was approved by the Ethics and Research Committees of the National Institute of Public Health and IMSS. Written informed consent was obtained from all participants’ mothers.

### 2.2. Added Sugars

AS intake from foods and beverages was estimated using interviewer-led 24 h dietary recalls collected at 6, 9, 12, 18, and 24 months. Recalls were administered on both weekdays and weekends, with approximately 20% conducted on weekend days. Although a second recall was obtained for about 10% of children at each age, only one recall per child was used in this analysis. The 24 h recall was obtained using the five-step multiple-pass method designed to reduce underreporting of dietary intake [14], adapted for the Mexican context [15]. Portion sizes were estimated using household measures. Breast milk intake was also quantified: when expressed breast milk was reported, the provided volume was used to calculate grams consumed; for direct breastfeeding, age-specific estimates of average intake per feeding episode were applied to derive breast milk intake [16,17]. Methodology for processing dietary information has been described elsewhere [18]. Estimation of AS was based on information from the Mexican Food Composition Database (BAM) [19], applying an algorithm adapted from the methodological approach proposed by Louie et al. [20], as modified by our research group [21]. Subsequently, tertiles were calculated to classify AS intake as low (0 g), medium (0.01–6.96 g), and high (>6.96 g). Although added sugar was initially examined as a continuous variable, tests indicated that associations were not linear. Tertiles provided meaningful distinctions between children with no added sugar intake, those consuming very small amounts, and those consuming around one teaspoon per day. Quartiles and finer categorizations were explored but resulted in sparse groups at several ages. In addition, foods and beverages reported in the 24 h recalls were classified according to their AS content using the food groups proposed and applied in the adaptation of Louie’s methodology [20].

### 2.3. Adiposity and Growth

Trained personnel obtained standardised measurements [22], following the Habicht protocol for anthropometric standardization [23]. Duplicate measurements of weight, length, and abdominal circumference, and triplicate measurements of skinfold thickness (triceps, biceps, subscapular, and suprailiac) were collected at birth and at 6, 9, 12, 18, and 24 months of age. For 170 infants, it was not possible to obtain birth measurements; therefore, these were retrieved from medical records. Infants were weighed in light clothing using a portable electronic pediatric scale (Tanita BABY MOMMY model 1582, Tokyo, Japan) with a precision of 10 g. Length was measured using a wooden infantometer (Schorr. Mexico City, Mexico) with a precision of 0.1 cm. Abdominal circumference was measured with a fibreglass measuring tape accurate to 0.1 cm. Skinfold thickness was measured using Lange calipers (from Los Angeles, CA, USA) to the nearest 1.0 mm. All measurements were averaged, and the sum of the four skinfolds was calculated, along with BMI-for-age and length-for-age Z scores using the World Health Organization growth standards for children aged 0–5 years [24].

### 2.4. Appetite and Satiety Indicators

At the infants’ 6-, 9-, 12-, 18-, and 24-month visits, the Child Eating Behaviour Questionnaire (CEBQ) [25] was administered to parents or primary caregivers. The CEBQ is a parent-reported subjective measure consisting of 35 items rated on a five-point Likert-type scale, with response options ranging from “never” to “always.” Higher scores reflect more pronounced expressions of each eating behaviour. It comprises eight subscales that characterize traits related to children’s eating behaviour, including: (1) food responsiveness, (2) enjoyment of food (in which higher scores generally reflect children’s appetite for food or desire to eat in response to food exposure, and these have been associated with obesity); (3) satiety responsiveness (a higher score represents a child’s ability to reduce food intake after eating in order to regulate energy intake and sensitivity to satiety cues); (4) slowness in eating (a high score “characterises a reduction in eating pace as a result of lack of enjoyment or interest in food”); (5) desire to drink (a high score reflects children’s desire to consume beverages); (6) food fussiness (a high score refers to the rejection of a substantial variety of familiar and new foods, leading to an inappropriate range of food intake); (7) emotional overeating; and (8) emotional undereating (a high score in these subscales reflects an increase or decrease in eating in response to negative emotions) [26,27].

For its use in the MAS-Lactancia population, the CEBQ was translated into Spanish and then back-translated into English to assess consistency and evaluate comprehension. The reliability of the Spanish-translated version of the instrument was calculated at 6, 9, 12, 18, and 24 months of age (publication in process), showing acceptable ranges for most subscales (α ≥ 0.60 to <0.80).

### 2.5. Breastfeeding Duration

Breastfeeding practices were assessed at 1, 3, 6, 9, 12, 18, and 24 months using a validated questionnaire following standard WHO status quo and recall methods, as detailed elsewhere [28]. Briefly, mothers reported breastfeeding initiation, type of milk consumed, feeding mode, frequency, and reasons for breast or bottle feeding, along with the introduction of foods and beverages. Breastfeeding during the first six months was classified according to WHO categories (exclusive, predominant, partial, or no breastfeeding), but for analysis exclusive breastfeeding (EBF) duration was grouped as <1 month, 1–3 months, and >3 to ≤6 months due to small numbers in other categories. Continued breastfeeding (CBF) from six months onward was defined as ongoing breast milk consumption at each follow-up assessment.

### 2.6. Covariates

To identify covariates for model adjustment, we constructed directed acyclic graphs considering both the prenatal and postnatal periods to determine the minimal sufficient adjustment set. The models were adjusted for maternal education, occupation, pregestational BMI, weight gain during pregnancy, child’s birthweight, energy intake (using the all-components model [29]), lactation modality, and the household wealth index. The wealth index was generated using principal component analysis and included variables related to housing conditions (housing type, construction materials, water and sanitation services), ownership of home appliances, electronics, and number of rooms. The methodology used to derive this index has been published elsewhere [30,31]. We also examined a sex-by-AS intake interaction term, which was not statistically significant.

### 2.7. Statistical Analysis

We described the general characteristics of the study population and evaluated potential selection bias by comparing participants included and excluded from the analysis. Categorical variables were summarised using frequencies, and continuous variables using means ± standard deviation (SD) or medians with interquartile ranges according to their distribution. Differences between included and excluded participants were assessed with chi-square tests for categorical variables, Student’s *t*-tests for normally distributed continuous variables, and Wilcoxon’s rank-sum test for non-normally distributed continuous variables.

To characterise AS intake at each timepoint, we estimated median daily AS intake and AS contribution to energy intake and calculated the mean contribution of the top 10 food groups to total AS intake. Bar graphs illustrating the contribution of each food group to total AS intake were generated with RStudio Version 2026.01.1 “Apple Blossom” [32] using the ggplot2 package [33].

For the association between AS intake and indicators of appetite, satiety, growth, and adiposity, we fitted linear mixed models with a random intercept at the child level to account for the correlation arising from having exposure and outcomes measured at multiple timepoints for each child, adjusting for covariates specified in the covariate section above (total observations: *n* = 823). For the all-components energy adjustment model, we included protein, lipid, and carbohydrate intake (excluding AS) in grams per day. Missing data were handled under the missing-at-random assumption, using all available observations. Model assumptions were evaluated using residual diagnostics, with no major violations detected. *p*-values for trend were calculated by modelling AS tertiles as a continuous variable.

As an additional exploratory analysis, we examined whether breastfeeding duration was associated with AS intake by fitting linear mixed-effects models with AS intake (g/day) as the outcome and breastfeeding duration (both EBF and CBF) as the exposures. EBF and CBF models were adjusted for maternal education, occupation, pregestational BMI, gestational weight gain, child’s birthweight, and household wealth index; the CBF model was additionally adjusted for EBF duration. All analyses were conducted using Stata version 19 (StataCorp, College Station, TX, USA). All tests were 2-tailed, with significance defined at the 5% level (alpha = 0.05).

## 3. Results

### 3.1. Participant Characteristics and Descriptive Statistics

Table 1 presents the general characteristics and AS intake of the study participants. Most mothers had at least 10 years of education, approximately half were formally employed, and 36% were classified in the “high” Household Wealth Index category. The average pregestational BMI was around 25 Kg/m^2^, and the median gestational weight gain was 9.2 Kg. About 48% of children were male, the mean birthweight was 3.1 Kg, and ~45% were exclusively breastfed for more than 3 months. As expected, median AS intake increased with age, reaching 19.4 g/day at 24 months, and AS accounted for 6% of total energy intake at the same age. When comparing included and excluded participants (due to missing data) (Appendix A), significant differences were observed in maternal education, with mothers of included participants having more years of schooling.

Descriptive statistics for anthropometric measures and child eating behaviours at 6, 12, and 24 months are presented in Table 2. Mean BMI-for-age Z scores remained close to the WHO reference median across all ages, while waist circumference increased steadily from 6 to 24 months. The sum of skinfolds decreased over time, and length-for-age Z scores were consistently below the WHO median, with the lowest values observed at 12 months. CEBQ subscales indicated age-related changes in eating behaviours. Food responsiveness, emotional over- and under-eating, and desire to drink increased with age, whereas enjoyment of food, satiety responsiveness, and slowness in eating remained relatively stable. Food fussiness was higher at 24 months than at earlier timepoints.

### 3.2. Association Between Breastfeeding Duration and Added Sugar Intake

Compared with infants exclusively breastfed for <1 month or not exclusively breastfed, those breastfed for >3 months had lower AS intake from 6 to 24 months of age (β = −3.59 g/day; 95% CI: −6.34, −0.84; *p* = 0.011) (Table 3). CBF was also associated with lower AS intake, with breastfed children consuming 9.78 g/day less than those not receiving breastmilk (95% CI: −13.20, −6.34; *p* < 0.001).

### 3.3. Food Group Contributions to Total Added Sugar Intake

**Figure 2:**
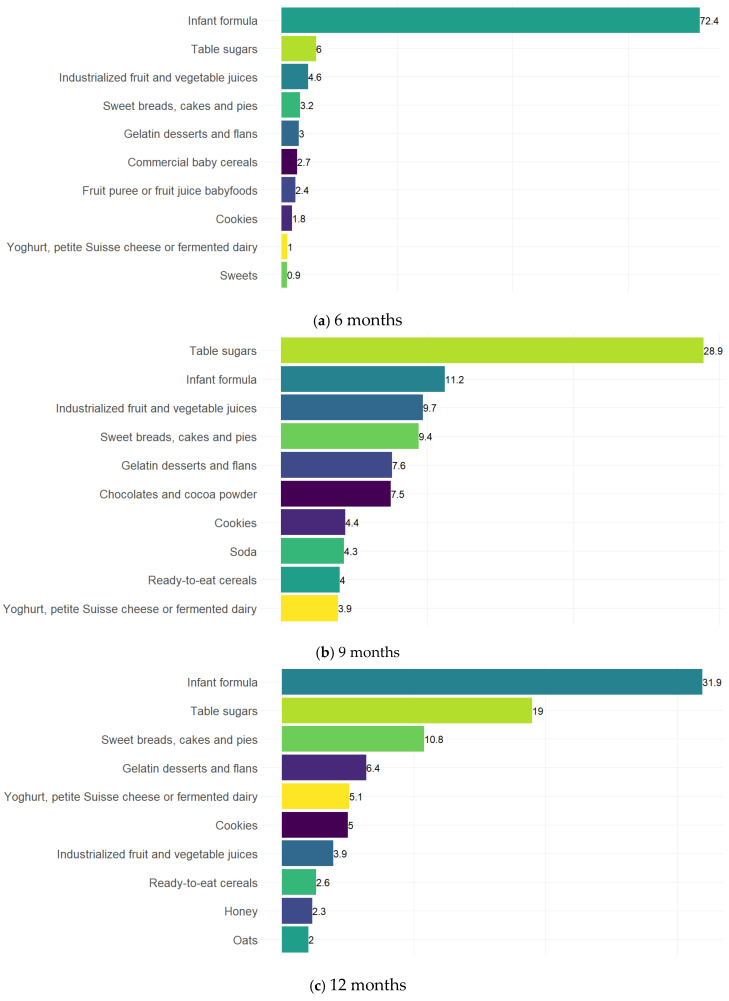

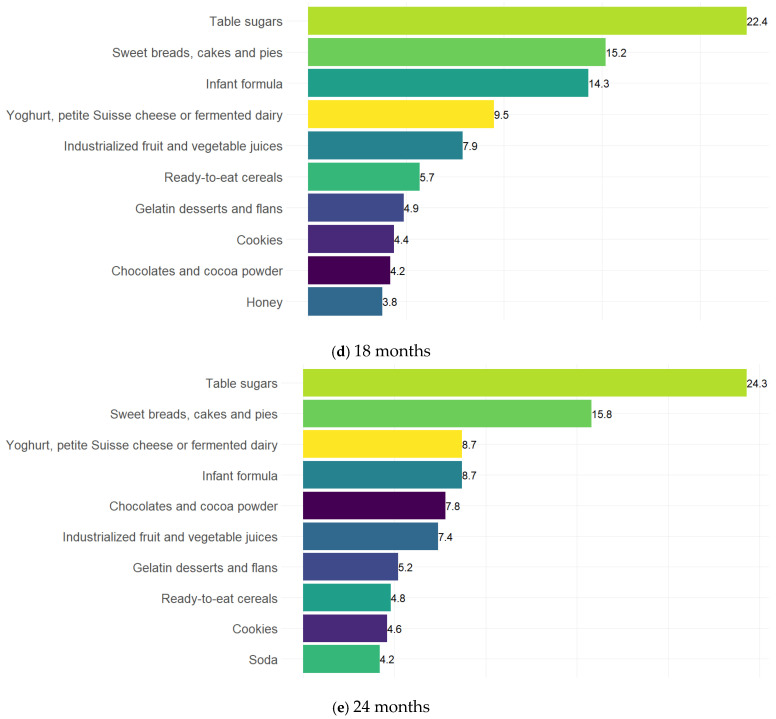
Top 10 Food Groups Contributing to Added Sugar Intake in Children Aged 6 to 24 Months, MAS-Lactancia cohort.

Figure 2 illustrates the top ten food groups contributing to AS intake across timepoints. At 6 months (Figure 2a), AS was overwhelmingly contributed by infant formula, which accounted for more than 70% of total intake. By 9 months (Figure 2b), table sugars became the leading source (28.9%), followed by infant formula (11.2%) and industrialised fruit and vegetable juices (~10%), with a growing contribution from sweet baked goods, gelatin desserts, chocolates, cookies, and soda. At 12 months (Figure 2c), infant formula again emerged as the main contributor (31.9%), followed by table sugars (19%), sweet breads, and gelatin desserts. At 18 months (Figure 2d), table sugars were once again the dominant source (22.4%), followed by sweet baked goods (15.2%) and infant formula (14.3%), with relevant contributions from yoghurt/fermented dairy and industrialised juices. At 24 months (Figure 2e), table sugars consistently remained the largest contributor (24.3%), followed by sweet baked goods (15.8%). Yoghurt/fermented dairy products, infant formula, chocolates, and industrialised juices also remained as relevant sources, alongside smaller contributions from gelatin desserts, cereals, cookies, and soda.

### 3.4. Associations Between Added Sugar Intake and Appetite and Satiety Indicators

The associations between added sugar (AS) intake and subjective indicators of appetite and satiety during the first 24 months of life are shown in Table 4. Higher AS intake was associated with greater emotional overeating (β = 0.58, 95% CI: 0.04, 1.12; *p* = 0.035), with a significant trend across intake categories (*p* trend = 0.033). High AS intake was also associated with greater food fussiness (β = 1.45, 95% CI: 0.38, 2.53; *p* = 0.008), while medium AS intake was positively associated with satiety responsiveness (β = 0.76, 95% CI: 0.01, 1.52; *p* = 0.048), with a marginally significant association observed for high intake. In contrast, food enjoyment tended to be lower among children with high AS intake (β = −0.60, 95% CI: −1.30, 0.09; *p* = 0.087). No meaningful associations were observed for the remaining subscales.

### 3.5. Association Between Added Sugar Intake and Child Growth and Adiposity

Table 5 shows the associations between AS intake and indicators of child growth and adiposity during the first 24 months of life. Children with high AS intake had approximately 2 cm greater waist circumference than those with low intake (β = 2.02 cm, 95% CI: 1.32, 2.73; *p* < 0.001), with a linear trend across categories (*p* trend < 0.001). High AS intake was also associated with lower linear growth (β = −0.17 Z score, 95% CI: −0.32, −0.01; *p* = 0.037; *p* trend = 0.034). No significant associations were observed for BMI-for-age Z scores and skinfold sum.

## 4. Discussion

Our findings indicate that infants exclusively breastfed for >3 months and with CBF had lower AS intake. Among children under 2 years of age, added sugar (AS) intakes exceeding 7 g/day were associated with eating behaviours such as greater emotional overeating and higher food fussiness. At this intake level, compared with no AS intake, children also exhibited lower linear growth and greater central adiposity. Additionally, AS intake increased rapidly over the first two years of life and was largely driven by the consumption of foods typically classified as ultra-processed—including commercial infant formulas—as well as the use of table sugars added during food preparation.

The contribution of AS to total energy intake increased steadily with age, rising from a mean of 0% at 6 months to 6% at 24 months. This is lower than what was reported by Rupérez et al. [34] in children under 4 years across several countries (ranged between 9.8% and 11.2%). This difference may reflect the younger age of our participants and the increasing presence of AS with age. It is also important to consider that participants from the MAS-Lactancia birth cohort received a breastfeeding counselling intervention designed to ensure an adequate number of breastfeeding children. Given that breastfeeding duration was associated with lower added sugar intake, this intervention may partly explain the observed differences. However, the pattern of food groups was broadly consistent with other studies. Their study identified fruit-based drinks and soft drinks as the main contributors to AS intake, followed by dairy products and sweet bakery items. Nonetheless, among the top contributors to AS intake across ages in our population were infant formulas and table sugars. The findings on the association between breastfeeding duration with lower added sugar intake are consistent with the results reported in studies conducted in the United States [35,36,37].

Evidence in children under two years of age remains limited. However, Kong et al. [38] documented the contribution of AS from commercial infant formulas, which accounted for 66% and 7% of total added sugar intake in infants and young children, respectively. Their study found a significant association between AS consumed through commercial infant formula and greater weight gain. Specifically, infants who were primarily formula-fed consumed nearly twice as much energy from added sugars compared with breastfed infants.

Regarding eating behaviours and AS intake, emotional eating has been described as a strategy to reduce stress, which is partly regulated by the hypothalamic–pituitary–adrenal (HPA) axis [39]. Consumption of sugar-containing foods can reduce HPA axis activity, producing short-term stress relief and increasing preference for comforting, palatable foods, and this cycle can reinforce emotional eating patterns over time [39]. Conversely, food fussiness has been linked to sensory sensitivity and lower reward responsiveness to food [40]. Moreover, having a higher preference for sweet tastes can make children more selective toward other foods and increase reliance on sweet items to ensure adequate intake. While our results may reflect early emerging traits related to appetite regulation, it is also plausible that parental responses play a role. Parents may be offering sweet foods to soothe children during episodes of distress or emotional reactivity, or they may use palatable foods to encourage eating in children perceived as fussy, and this can further reinforce emotional overconsumption of sweet foods and perpetuate a cycle of selectivity and preference for sweetness. Such bidirectional dynamics between child behaviour and parental feeding practices have been described in the literature [41,42].

Regarding child growth and adiposity, differences of this magnitude (+2 cm in abdominal circumference and −0.17 Z in length-for-rage) can be meaningful in children under two years of age. For context, a previous study showed that a one SD increase in abdominal circumference (approximately 3 cm at 36 months) was associated with measurable differences in blood pressure [43]. Even a −0.17 Z score difference in length-for-age should be considered relevant given that the first 1000 days represent a critical window for growth, during which most linear growth faltering occurs in low and middle-income countries [44]. This is particularly important in our cohort, where the population mean for length-for-age was consistently below zero (−0.80 Z at 6 months, −1.15 Z at 12 months, and −1.01 Z at 24 months), indicating a general vulnerability to impaired linear growth. Potential pathways could include nutrient displacement (with high energy provision), effects of high glycemic load on insulin–IGF-1 signaling, and alterations in gut microbiota. Similar to our results, Herbst et al. [45] showed that an increase in AS consumption between ages one and two years was associated with higher BMI at seven years of age. Other authors have also documented that higher intake of AS in the early years period is associated with an increased risk of obesity [38]. The findings from these studies are consistent with our results, which indicate that higher AS intake during the first two years of life is associated with greater abdominal circumference (or abdominal adiposity). However, in contrast to Herbst et al. [45] and others, we did not observe differences in BMI or in the sum of skinfolds at two years of age. Our findings also showed that higher AS intake from birth to 24 months of age was associated with lower linear growth (length-for-age). We did not identify previous studies reporting similar results. However, the risks associated with consumption of foods high in AS are well recognized, as these foods often provide “empty calories” and may compromise adequate growth and development in young children by displacing nutrient-dense foods in the diet [46].

Given this association, we conducted additional analyses to explore potential mechanisms through which this relationship might arise. Using mixed-effects models, we examined the relationship between AS intake and overall diet quality. We found that higher AS intake during the first 24 months of life was significantly associated with a greater contribution of unhealthy indicator foods to total dietary intake. We also evaluated vegetable consumption and observed that lower intake of added sugars was related to higher vegetable intake at 6 and 24 months of age, with statistically significant differences.

This study has several limitations. First is the loss of a substantial number of participants from the original sample due to decreasing response rates over the course of the cohort follow-up. Nonetheless, comparing differences in general characteristics between those included and excluded in the analyses showed differences only in maternal education level: a higher proportion of mothers with higher education was observed in included participants (Appendix A). Second, dietary data were collected using 24 h recalls, which are widely used in nutrition research but are subject to recall bias and misreporting. Such limitations may lead to underestimation of added sugar intake. However, the structured five-step multiple-pass methodology used in the dietary recalls mitigates these potential sources of error. In addition, 24 h recalls remain the most widely recommended method for dietary assessment in epidemiologic studies because they provide detailed intake data with relatively low respondent burden. Moreover, the calculation of added sugars in the food composition database was indirect. We estimated AS content adapting the method proposed by Louie et al. [20], which has been shown to be the most detailed and comprehensive approach for this purpose, even when compared with methodologies proposed by the Pan American Health Organization for determining added sugars [47]. Additionally, according to the regulatory framework, the estimation of added sugars from infant formulas may be underestimated. When labels explicitly reported the amount of added sugar, we used that value directly. When added sugars were not reported, we estimated them by subtracting the amount of lactose from the total carbohydrate content, as lactose was considered a naturally-occurring sugar. However, due to the lower lactose content in cow’s milk, lactose is also added as an ingredient in infant formulas [48].

Another aspect to consider is the use of subjective indicators to assess the different constructs related to eating behaviours concerning appetite and satiety. Although these measures are subjective, the instrument used has been validated for this population, with only modest internal consistency observed at 6 months of age (publication in process). This was expected, as this is a developmental stage in which complementary feeding is just beginning, making it more difficult for parents to identify food-related behaviours and accurately perceive appetite and satiety cues. It is also important to note that evidence regarding these indicators in this age group remains limited, and to the best of our knowledge, this study is among the first to document these relationships using this scale during this early developmental period. It is also worth noting that an association between added sugar intake and both suboptimal growth and central adiposity was observed even within this population, which had a longer-than-average breastfeeding duration and relatively low overall added sugar intake. Although cultural and socioeconomic factors may shape the specific sources and patterns of added sugar exposure across settings, we consider that the biological mechanisms through which added sugars influence growth and eating behaviours are unlikely to differ between populations. Finally, the prospective design strengthens temporal interpretation, but causal inference remains limited, and some degree of reverse causation cannot be fully ruled out.

One of the major strengths of this study is its prospective birth cohort design, focused on characterising key feeding stages, which provides unique value by capturing data at several relevant ages throughout early childhood. This design offers a comprehensive perspective on feeding practices and exposures during a rapid-changing period, enabling assessment of risk patterns [13]. Another important strength is the method used to assess infant feeding: 24 h dietary recalls, which are recommended by international organizations as the most appropriate approach for evaluating dietary intake in infants and young children [49]. Finally, anthropometric and dietary data were collected by trained and standardised personnel, ensuring high-quality measurements.

These findings are consistent with global policy frameworks, including WHO complementary feeding guidelines [50], and reinforce the importance of adhering to recommendations that advise against the inclusion of added sugars in foods and beverages for children under two years of age. Early exposure to added sugars may contribute to suboptimal growth, increased central adiposity, and non-optimal eating patterns that tend to persist later in life. Beyond table sugar, the main contributors to added sugar intake in this cohort were foods commonly classified as ultra-processed—most notably infant formulas, fruit and vegetable juices, and sweet baked goods—with intake increasing progressively with age.

In Mexico, regulatory oversight of added sugars in commercial infant formulas remains limited [51]. These products are exempt from the front-of-pack warning labels established under NOM-051 [52], as they fall under formula-specific regulations with their own nutritional criteria. However, current standards do not explicitly address added sugars permitted in infant formulas, creating an important regulatory gap given the high reliance on these products during early life.

## 5. Conclusions

Overall, our findings highlight the urgency of strengthening national and international policies aimed at reducing unnecessary added sugar exposure during infancy and early childhood. Taken together, these findings have direct implications for infant feeding guidelines, the regulation of commercial baby foods and infant formulas, and breastfeeding promotion efforts. They reinforce the need to avoid added sugars during the first two years of life, support stronger regulatory oversight of added sugars in commercial products, and highlight breastfeeding as a key protective practice against early sugar exposure. The policy recommendation box below summarises key recommendations. Further research across varied populations is needed to confirm and contextualise these observations globally, to inform more comprehensive regulatory frameworks that protect infants from excessive sugar intake during this critical developmental window, and to evaluate strategies for reducing added sugar exposure during infancy, as well as to examine potential effects on linear growth trajectories into later childhood. Policy recommendations:

- Strengthen programmes that support exclusive and continued breastfeeding.
- Establish clear regulatory limits for added sugars in commercial infant formulas and require transparent carbohydrate-source labelling.
- Implement stricter marketing controls for commercial baby foods and apply WHO Code for breast milk substitute marketing
- Strengthen public health messaging on added sugar avoidance for babies and infants, including juice drinks and desserts
- Establish evidence surrounding potential benefits of applying front-of-pack warning labels to commercial baby foods and infant formula products containing added sugars
- Integrate guidance that encourages nutrient-dense foods and discourages table sugar addition during food preparation.

## Figures and Tables

**Table 1:** General characteristics and added sugar intake of study participants.

Maternal Characteristics	Subsample *N* = 248
Education, *n* (%)	
≤9 years	61 (24.6)
10–12 years	101 (40.7)
≥13 years	86 (34.7)
Household Wealth Index, *n* (%)	
Low	78 (31.5)
Medium	81 (32.7)
High	89 (35.9)
Occupation, *n* (%)	
Not employed/homemaker	80 (32.3)
Informal employment or student	37 (14.9)
Formal employment	131 (52.8)
Pregestational BMI (Kg/m^2^), μ ± D.E.	24.8 ± 4.3
Gestational weight gain (Kg), p50 (p25, p75).	9.2 (5.7, 12.1)
**Child’s characteristics**	
Sex, *n* (%)	
Male vs. Female	118 (47.6)
Birthweight (Kg), μ ± D.E.	3.1 ± 0.5
Exclusive breastfeeding duration, *n* (%)	
<1 month or no exclusive breastfeeding	85 (34.3)
1–3 months	53 (21.4)
>3 months	110 (44.4)
Added sugar intake (g/day), p50 (p25, p75)	
6 months	0.0 (0.0, 1.8)
9 months	0.0 (0.0, 6.3)
12 months	5.9 (1.5, 16.6)
18 months	10.4 (3.5, 21.4)
24 months	19.4 (6.6, 37.5)
Added sugar contribution to total energy intake (%), p50 (p25, p75)	
6 months	0.0 (0.0, 0.8)
9 months	0.1 (0.0, 5.1)
12 months	2.6 (0.5, 6.4)
18 months	3.8 (1.6, 7.7)
24 months	6.0 (2.5, 11.1)

**Table 2:** Child Eating Behaviour (CEBQ) Subscales and Anthropometric Measures (Growth and Adiposity) at 6, 12, and 24 Months.

	Assessment Times		
	6 Months	12 Months	24 Months
**Adiposity and growth, μ ± D.E**			
BMI-for-age (Z score)	−0.01 ± 1.04	0.12 ± 1.12	0.02 ± 0.94
Waist circumference (cm)	42.38 ± 2.76	43.53 ± 3.01	45.59 ± 2.84
Skinfold sum (mm)	28.84 ± 6.22	26.86 ± 6.24	22.41 ± 5.74
Length-for-age (Z score)	−0.80 ± 0.96	−1.15 ± 1.14	−1.01 ± 0.90
**Child Eating Behaviour Subscales, p50 (p25, p75)**			
Food responsiveness	10 (7, 14)	11 (9, 14)	12 (8, 16)
Emotional over-eating	4 (4, 7)	6 (4, 8)	7 (4, 8)
Enjoyment of food	18 (15.5, 20)	18 (15, 20)	17 (15, 20)
Desire to drink	6 (4, 9)	8 (5, 10)	9 (6, 12)
Satiety responsiveness	13 (11, 15)	13 (11, 15)	13 (10, 15)
Slowness in eating	12 (9, 14)	12 (10, 14)	11 (10, 13)
Emotional under-eating	10 (8, 14)	12 (9, 16)	12.5 (9, 16)
Food fussiness	12 (9, 16)	12 (10, 15)	15 (12, 18)

**Table 3:** Association between Breastfeeding Duration and Added Sugar Intake in the first 24 months of age.

Breastfeeding	Added Sugar Intake (g/Day)		
Exclusive breastfeeding	β	95% CI	*p* value
<1 month or no exclusive breastfeeding	Ref.		
1–3 months	−2.80	−6.27, 0.68	0.114
>3 months	−3.59	−6.34, −0.84	0.011
Continued breastfeeding			
Yes vs. No	−9.78	−13.20, −6.34	<0.001

Linear mixed models were used adjusted for maternal education, occupation, pregestational BMI, weight gain during pregnancy, child’s birthweight, and the household wealth index. Continued breastfeeding model was additionally adjusted for exclusive breastfeeding duration.

**Table 4:** Associations between Added Sugar Intake and Appetite and Satiety Indicators in the first 24 months of age.

	Child Eating Behaviour Subscales											
	Food Responsiveness			Emotional Over-Eating			Food Enjoyment			Desire to Drink		
	β	95% CI	*p* Value	β	95% CI	*p* Value	β	95% CI	*p* Value	β	95% CI	*p* Value
Added Sugar Intake												
Low (0 g)	Ref.			Ref.			Ref.			Ref.		
Medium (0.01–6.96 g)	−0.35	−1.30, 0.60	0.472	0.15	−0.35, 0.65	0.555	−0.13	−0.77, 0.51	0.696	−0.28	−0.98, 0.42	0.431
High (>6.96 g)	−0.09	−0.93, 1.12	0.857	0.58	0.04, 1.12	0.035	−0.60	−1.30, 0.09	0.087	0.46	−0.30, 1.21	0.235
*p* for tendency			0.829			0.033			0.083			0.213
	Satiety Responsiveness			Slowness in Eating			Emotional Under-Eating			Food Fussiness		
	β	95% CI	*p* value	β	95% CI	*p* value	β	95% CI	*p* value	β	95% CI	*p* value
Added Sugar Intake												
Low (0 g)	Ref.			Ref.			Ref.			Ref.		
Medium (0.01–6.96 g)	0.76	0.01, 1.52	0.048	0.11	−0.54, 0.76	0.737	0.20	−0.69, 1.09	0.657	0.47	−0.53, 1.48	0.357
High (>6.96 g)	0.77	−0.04, 1.58	0.063	0.13	−0.57, 0.82	0.715	0.64	−0.32, 1.60	0.189	1.45	0.38, 2.53	0.008
*p* for tendency			0.069			0.719			0.185			0.008

Linear mixed models were adjusted for maternal education, occupation, pregestational BMI, weight gain during pregnancy, child’s birthweight, energy intake, lactation modality, and the household wealth index.

**Table 5:** Association between Added Sugar Intake and Child Growth and Adiposity in the first 24 months of age.

	Child Growth and Adiposity											
	BMI-for-Age (Z Score)			Length-for-Age (Z Score)			Waist Circumference (cm)			Skinfold Sum (mm)		
	β	95% CI	*p* Value	β	95% CI	*p* Value	β	95% CI	*p* Value	β	95% CI	*p* Value
Added Sugar Intake												
Low (0 g)	Ref.			Ref.			Ref.			Ref.		
Medium (0.01–6.96 g)	−0.04	−0.19, 0.11	0.583	−0.09	−0.23, 0.04	0.183	1.30	0.67, 1.93	<0.001	0.26	−0.84, 1.37	0.643
High (>6.96 g)	−0.03	−0.20, 0.13	0.686	−0.17	−0.32, −0.01	0.037	2.02	1.32, 2.73	<0.001	−0.19	−1.44, 1.07	0.771
*p* for tendency			0.654			0.034			<0.001			0.832

Linear mixed models were used adjusted for maternal education, occupation, pregestational BMI, weight gain during pregnancy, child’s birthweight, energy intake, lactation modality, and the household wealth index.

## Data Availability

Data available on request due to restrictions (due to ethical concerns regarding personal information from children).

## Abbreviations

The following abbreviations are used in this manuscript:

AS	Added sugars
BMI	Body mass index
BMI-for-age Z score	BMI Z score relative to WHO standards
ZLA	Length-for-age Z score
CEBQ	Child Eating Behaviour Questionnaire
EBF	Exclusive breastfeeding
CBF	Continued breastfeeding
FTO	Fat mass and obesity-associated gene
MAS-Lactancia	Name of the birth cohort
BAM	Mexican food composition database
ENSANUT	National Health and Nutrition Survey
IMSS	Mexican Social Security Institute
WHO	World Health Organization
CONACYT	National Council of Science and Technology

## Appendix A

**Table A1:** General Characteristics of Included and Excluded Participants from the MAS-Lactancia Birth Cohort.

	Included *N* = 248 (21.2%)	Excluded *N* = 921 (78.8%)	*p* Value
*Maternal Characteristics*			
Education, *n* (%)			
≤9 years	61 (24.6)	261 (28.8)	0.009
10–12 years	101 (40.7)	420 (46.3)	
≥13 years	86 (34.7)	226 (24.9)	
Missings		14	
Household Wealth Index, *n* (%)			
Low	78 (31.5)	278 (33.9)	0.581
Medium	81 (32.7)	276 (33.6)	
High	89 (35.9)	267 (32.5)	
Missings		100	
Occupation, *n* (%)			
Not employed/homemaker	80 (32.3)	338 (37.3)	0.222
Informal employment or student	37 (14.9)	107 (11.8)	
Formal employment	131 (52.8)	462 (50.9)	
Missings		14	
Pregestational BMI (Kg/m^2^), μ ± D.E.	24.8 ± 4.3	24.9 ± 4.5	0.499
Missings		230	
Gestational weigth gain (Kg), p50 (p25, p75).	9.2 (5.7, 12.1)	9.1 (5.5, 12.4)	0.621
Missings		710	
**Child’s characteristics**			
Sex, *n* (%)			
Male vs. Female	118 (47.6)	242 (51.9)	0.268
Missings, n		455	
Birthweight (Kg), μ ± D.E.	3.1 ± 0.5	3.1 ± 0.4	0.499
Missings		455	

BMI: Body Mass Index; *p* values were calculated using Pearson’s χ^2^ test for categorical variables; *t*-tests for variables reported as mean ± SD; and Wilcoxon rank-sum tests for variables reported as median and percentiles.
